# Intelligent RF-Based Gesture Input Devices Implemented Using e-Textiles †

**DOI:** 10.3390/s17020219

**Published:** 2017-01-24

**Authors:** Dana Hughes, Halley Profita, Sarah Radzihovsky, Nikolaus Correll

**Affiliations:** 1 Department of Computer Science, University of Colorado Boulder, Boulder, CO 80309, USA; halley.profita@colorado.edu; 2 Department of Electrical Engineering, Stanford University, Stanford, CA 94305, USA; sradzi13@stanford.edu

**Keywords:** e-textiles, wearable sensors, robotic materials

## Abstract

We present an radio-frequency (RF)-based approach to gesture detection and recognition, using e-textile versions of common transmission lines used in microwave circuits. This approach allows for easy fabrication of input swatches that can detect a continuum of finger positions and similarly basic gestures, using a single measurement line. We demonstrate that the swatches can perform gesture detection when under thin layers of cloth or when weatherproofed, providing a high level of versatility not present with other types of approaches. Additionally, using small convolutional neural networks, low-level gestures can be identified with a high level of accuracy using a small, inexpensive microcontroller, allowing for an intelligent fabric that reports only gestures of interest, rather than a simple sensor requiring constant surveillance from an external computing device. The resulting e-textile smart composite has applications in controlling wearable devices by providing a simple, eyes-free mechanism to input simple gestures.

## 1. Introduction

The development of conductive fabrics and threads (e-textiles) has allowed for the seamless integration of complex circuits into garments. These forms of technology continue to grow in both the commercial and research sectors. The combination of e-textiles and other conductive materials with physically small circuit components provides opportunities for flexible, textile-based on-body input devices to be directly integrated into garments. Specifically, e-textile widgets can mimic traditional graphical user interface widgets, such as buttons, jog-wheels, slides and keyboards [1,2,3], as well as provide novel modes of interaction [4]. These devices involve combining one or more e-textile sensing components (swatches) and a microcontroller circuit to measure samples from the swatches, such as LilyPad [5].

**Figure 1:**
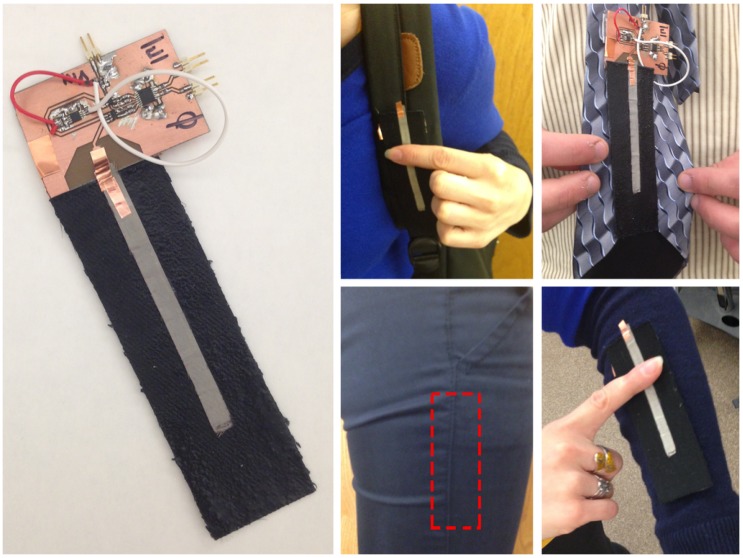
Prototype RF-based microstrip swatch and reflectometer circuit. Possible placements and applications are shown. From [8].

E-textile input devices usually rely on measuring variations in either capacitance [6], resistance [7] or a combination of the two [2,3] of a wearable circuit component. These forms of interface may require direct skin contact with the conductive fabric, especially with resistance-based or circuit completion-based approaches, or may be prone to accidental triggering. Industrial advances in wireless and cell phone devices have provided inexpensive, robust and readily-available mixed microwave integrated circuits (MMIC), which allow for the simple realization of RF- and microwave-based sensing. In this paper, we present an approach for RF-based sensing devices using a minimal number of MMIC components and easily constructed e-textile interfaces. This paper extends prior work, SwitchBack, a prototype device shown in Figure 1 [8]. RF-based e-textile sensors have several advantages over capacitive and resistive approaches. Direct skin contact is not required; this allows swatches to be weatherproofed, such as being embedded in silicone rubber (e.g., EcoFlex [9]), or to be located under a layer of fabric in cases where the garment design makes a visible sensor non-ideal. The swatch allows measuring finger position along a continuum of positions using only a single electrical connection, while similar capacitive devices measure a discrete number of finger position using multiple electrical connections.

The measurement circuit for RF-based swatches generates analog voltage signals. Small, inexpensive microcontrollers with analog-to-digital converters (ADCs) can be incorporated into the MMIC circuit to convert and process these signals. In addition, the microcontroller provides a platform that can be used to implement a classification system, provided the classification model does not exceed the memory or computational requirements of the microcontroller. By implementing such a classifier, the swatches provide a means of implementing intelligent sensing into fabric materials; the fabric itself detects and processes tactile interactions and only communicates events of interest to external computing devices. This approach may be viewed in the context of robotic materials, which describes the tight coupling between sensing and computing aspects and an underlying physical material [10].

The remainder of the paper is organized as follows: Section 2 provides the background and related work. The theoretical foundations for the sensors are given in Section 3, which are based on transmission line theory. Simulated results of the different types of transmission lines are given in Section 4 and provide the reasoning for using a microstrip sensor. Section 6 describes an approach to classifying gestures performed on the sensor. Experiments are described in Section 7, which validate the classification approach. Section 8 provides a discussion of the results and concludes the paper.

## 2. Related Work

Post and Orth explored e-textile interfaces by embroidering conductive thread into clothing [1]. A number of e-textile interfaces have since been devised that use capacitive [6,11,12,13], resistive [7], circuit completion [4] or hybrid resistive-capacitive [2,3] sensing methods for implementation. These interfaces permit touch input and are conducive for wearable forms of technology, as they can be directly sewn into garments. Each method offers particular advantages (resistive yields direct touching, capacitive enables hovering activation and the hybrid approach addresses issues with resistive or capacitive sensing alone). However, they also face a number of limitations. In the case of the hybrid sensing method, often elaborate interfaces need to be constructed to avoid shorting and involuntary activation. Capacitive sensing is often non-discriminatory with respect to the conducting agent, lending to false triggering. Resistive surfaces offer closed-circuit solutions ideal for discrete touch, but localizing triggers usually require the integration of numerous leads. This applies to all of the sensing methods mentioned, as these interfaces fall short in their ability to arbitrarily assess multipoint touch or continuous input without extensive lead construction, a huge fabrication challenge. These interfaces are also susceptible to malfunctioning if exposed to the elements.

Wimmer and Baudish have recently introduced a family of single- and two-wire sensors capable of measuring the position of a touch using time domain reflectometry (TDR) [14], a measurement technique where an electrical pulse is injected into a transmission line, and partial reflections (echos) of the pulse indicate the position at discontinuities in the line. While TDR was first developed to detect flaws in transmission lines, the approach can be used to detect and localize changes in the environment surrounding the line. For example, Sun et al. designed a coaxial cable to be embedded in concrete structures to determine the position and size of cracks [15].

e-Textiles have been explored recently for wearable antennas and RFID tags [16]. Wearable fabric antennas have been developed for several applications over a wide range of operation frequencies. Simple patch antennas for WLAN and Bluetooth communication (2.4 GHz–2.5 GHz) are described in [17]. An ultra-wideband antenna operating in the range of 3 GHz–20 GHz has been realized on a 6 cm × 6 cm fabric substrate for medical purposes [18]. Embroidered dipole antennas provide simple wearable RFID tags, operating at 800 MHz–1 GHz, which can be read in a range of up to 7 m [19]. Additionally, the transmission line properties for twisted pair lines made from conductive thread have demonstrated that e-textiles are suitable for transmitting signals at frequencies up to 1.2 GHz over a length of 10 cm [20].

In SwitchBack [8], an eyes-free, one-handed interface capable of discrete and multi-directional continuous on-body input was presented. The methods reviewed above [2,3,4,6] rely on discrete sensors, requiring an increased number of measurements and computational cost for similar resolution. SwitchBack is both fabrication simplistic and competent when either conspicuously or inconspicuously embedded in clothing. Using RF also allows SwitchBack to be weatherproofed (limiting susceptibility to the elements), allows easy detection of multiple types of input gestures (e.g., swiping, discrete touches) and is more robust to accidental triggering. The design of wearable antennas for airwaves, mobile telephone and WiFi network communications in [21,22] has shown the feasibility of using textiles for body-worn antennas and demonstrates that many fabrics are low-loss and robust to moisture and washing. Like the TDR approach in [14], our approach relies on changes to transmission line properties. However, our measurements are performed in the frequency domain, whereas TDR measurements are performed in the time domain. We measure a complex (magnitude and phase) reflection coefficient at the input of the transmission line using a continuous, single-frequency RF signal. There are several tradeoffs between the TDR approach and ours. TDR measurements suffer from multiple echos, which may cause erroneous measurements. In the frequency domain, each multiple reflection simply contributes to the measured steady-state reflected signal [23]. Determining the position of a touch using TDR involves peak detection, which involves calculating zero-crossing points of the derivative of the measurement. Using our approach, touch position has a near linear relationship with the phase of the reflection coefficient, making detecting and locating a touch much simpler. Unlike our approach, TDR is designed for use with very long transmission lines. In our approach, the length of the transmission line is limited by the frequency of the transmitted wave, in order to ensure a unique measurement for any touch location. Despite this limitation, the frequency range of our circuit components (100 MHz–2.7 GHz) allows for textile interfaces up to 75 cm in length, suitable for most wearable sensors.

The tight integration of sensing and computation with material geometry and physical properties, such as described in this paper, enables a new class of smart composites known as robotic materials [10]. Arranged into arrays and communicating with their local neighbors here, the input device described here could serve as a building block of a larger-scale sensing fabric, similar to tactile sensing skin described in [24], and enable entire garments or equipment covering to perform the analysis of complex gestures [25,26] or perform other distributed machine learning tasks [27], possibly allowing such materials to learn intelligent behavior based on external stimuli.

## 3. Approach

The gesture input devices described in this paper are based on the behavior of a short-circuited stub, a short length of transmission line with one end short-circuited, driven by a radio-frequency (RF) signal operating at a frequency in the range of several hundred kHz to several GHz. At these frequencies, the wavelength of the signal is such that the phase of the signal varies significantly over the length of the transmission line. The input impedance measured at the input (i.e., non-shorted end) of the line is based on the frequency of the signal, the length of the line and the characteristic impedance of the line [23]. The characteristic impedance of the line is sensitive to variations in the geometry and material properties of the line.

### 3.1. Transmission Line Theory

**Figure 2:**
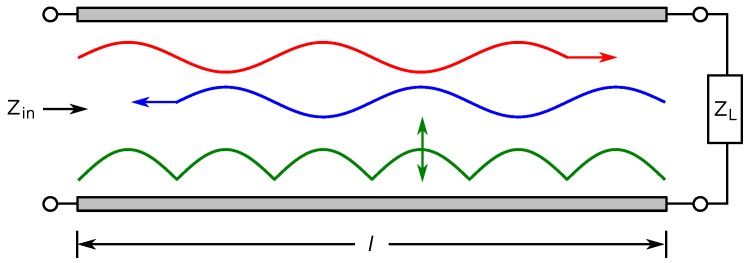
Incident signal (**red**), traveling towards the load, and the reflected signal (**blue**), due to impedance mismatch. The combination of the two waves results in a standing wave (**green**).

**Figure 3:**
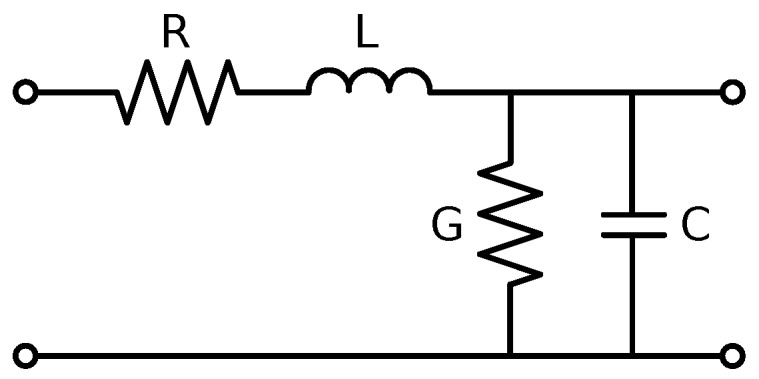
Infinitesimal transmission line.

A transmission line is schematically represented as a two-wire line of length *l*, shown in Figure 2. The line can be treated as an infinite series of infinitesimal transmission lines, which models the per-unit-length resistance, *R*, and inductance, *L*, of the conductors and the capacitance, *C*, and conductance, *G*, of the dielectric separating the conductors, as shown in Figure 3. From these values, the propagation constant, *γ*, and characteristic impedance, $Z_{0}$, of the line can be calculated using the telegrapher’s equations:

(1) $$\gamma =\sqrt{\left(R+j\omega L)(G+j\omega C\right)}=\alpha +j\beta$$

(2) $$Z_{0}=\sqrt{\frac{R+j\omega L}{G+j\omega C}}$$

where *ω* is the frequency of the electrical signal on the line and *j* is the imaginary unit.

The transmission line shown in Figure 2 is terminated by an arbitrary load impedance, $Z_{L}$. An incident RF voltage signal, $V_{f}\left(z\right)=V^{+}e^{-\gamma z}$, propagates down the line from the input. At the load end of the line (z=l), the signal is partially or fully reflected due to mismatch between the load impedance and characteristic impedance of the line, resulting in a reflected signal, $V_{r}\left(z\right)=V^{-}e^{\gamma z}$. The relationship between the incident and reflected signals is given by the reflection coefficient, Γ, which is the ratio of the incident and reflected signal evaluated at the load end of the signal, which is calculated from the load impedance and the characteristic impedance:

(3) $$\Gamma ={\left.\frac{V_{r}\left(z\right)}{V_{i}\left(z\right)}\right|}_{z=l}=\frac{Z_{L}-Z_{0}}{Z_{L}+Z_{0}}$$

The combination of the incident and reflected signals form a standing wave on the line. The input impedance of the line at an arbitrary point, $Z_{in}\left(z\right)$, can be calculated as the ratio of the total voltage and current at that point on the line. For the transmission line in Figure 2, the input impedance of the line is given by:

(4) $$Z_{in}=Z_{0}\frac{Z_{L}+Z_{0}tanh\left(\gamma l\right)}{Z_{0}+Z_{L}tanh\left(\gamma l\right)}$$

Details of this derivation are available in [23].

**Figure 4:**
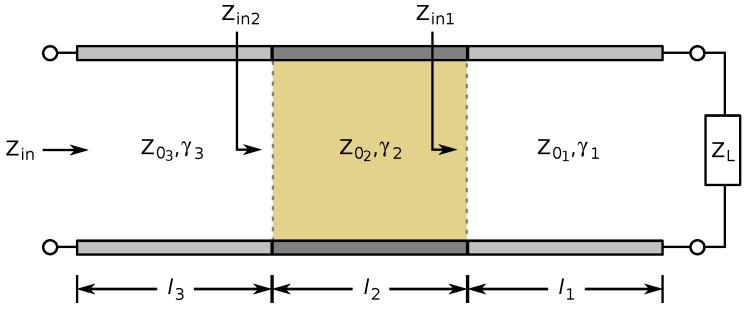
Approach to calculating the input impedance of a transmission line with multiple sections of different characteristic impedances.

The input impedance of more complex transmission lines can be calculated by recursively applying Equation (4). In this paper, transmission lines consist of multiple sections with different characteristic impedances, based on whether a section is touched or not. A model of this situation is given in Figure 4. The input impedance is calculated for the section closest to the load impedance, which is used as the load impedance to calculate the input impedance of the next section of the transmission line. This is repeated until the input impedance of the transmission line is determined.

The characteristic impedance, $Z_{0}$, is determined by the cross-sectional geometry of the transmission line. The impedance has been derived and analyzed for several common transmission lines in the literature. For this investigation, we consider e-textile transmission lines based on coaxial, microstrip and stripline, as e-textile versions of these are easily fabricated, and touching or applying pressure to the resulting line results in significant changes to the characteristic impedance. Furthermore, we only model the propagation of transverse electromagnetic (TEM) or quasi-TEM waves, as higher order modes of propagation are typically evanescent and will not significantly alter the measured reflected wave.

#### 3.1.1. Coaxial

**Figure 5:**
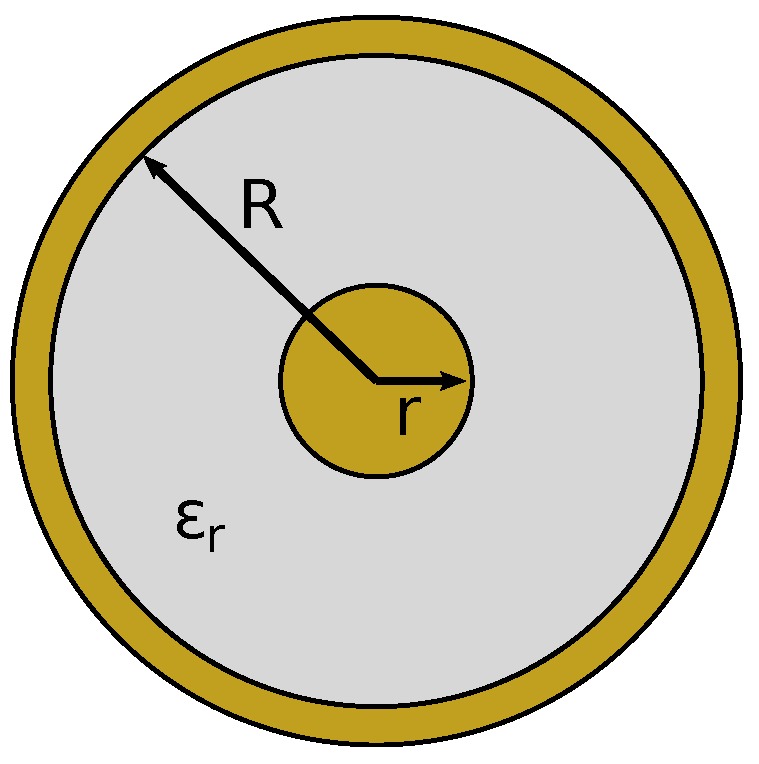
Cross-section of a coax transmission line.

**Figure 6:**
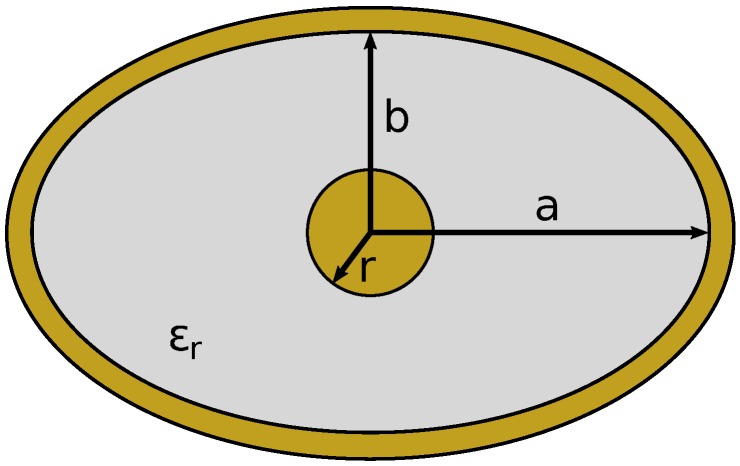
Cross-section of an elliptical coax transmission line.

A coaxial line consists of an inner conductor surrounded by an insulating layer and an outer conducting sheath. To simplify analysis and construction, circular cross-sections are common for coaxial lines, as shown in Figure 5, which consist of an inner conductor of radius *r*; the outer conductor has a radius *R*, and the insulator has a relative (to free space) permittivity of $\epsilon_{r}$. Elliptical cross-sections, such as shown in Figure 6, have also been analyzed [28], where the inner conductor has a circular cross-section of radius *r*, and the outer conductor is an ellipse with semi-major and semi-minor axes of *a* and *b*, respectively. Elliptical cross-sections are of interest in this investigation, as they may be viewed as a distortion of the circular cross-section due to warping from pinching or other tactile interaction.

The characteristic impedance and propagation constant of a circular cross-section coaxial line are available in the literature [23]. The characteristic impedance for coaxial lines with non-circular outer conductors, such as in Figure 6, can be calculated using an approximate graphical method and equivalent eccentric coaxial lines [28,29].

#### 3.1.2. Microstrip

**Figure 7:**
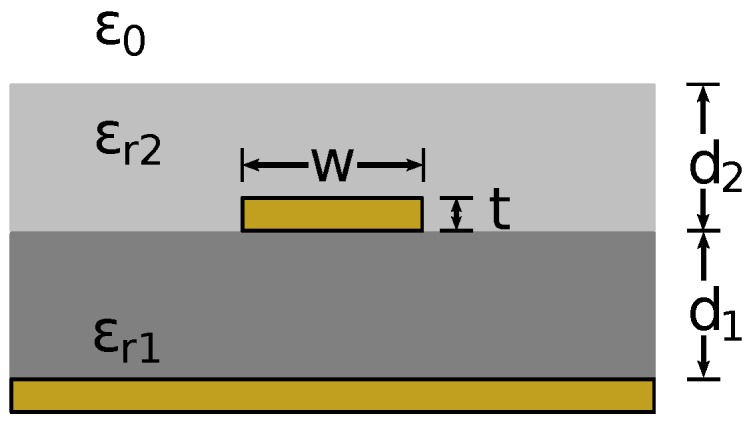
Cross-section of a microstrip transmission line with an optional dielectric superstrate.

A microstrip is a transmission line consisting of a conductive strip separated from a ground plane using a dielectric substrate, as shown in Figure 7. The conductive strip has width *w* and thickness *t*, and the dielectric has thickness $d_{1}$ and relative permittivity $\epsilon_{r1}$. The microstrip can optionally be covered with a dielectric layer with a relative permittivity of $\epsilon_{r2}$ and thickness $d_{2}$, such as when touched with a finger. For this investigation, the dielectric layer is assumed to be lossy and thick enough to be considered a half-space.

The characteristic impedance and propagation constant for the case where there is no dielectric cover is well established and is given in [23,30]. For the case of a microstrip covered with a dielectric, the characteristic impedance can be calculated using a conformal mapping method [31].

#### 3.1.3. Stripline

**Figure 8:**
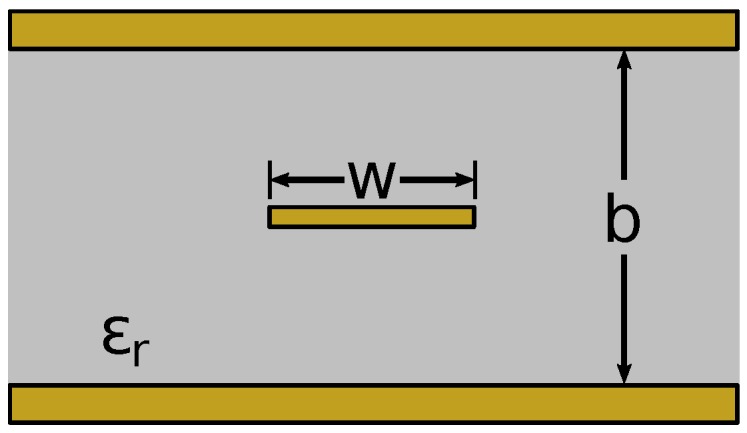
Cross-section of a stripline transmission line.

A stripline is a transmission line similar to a microstrip where a ground plane is present above and below the conductive strip, as shown in Figure 8. The conductive strip is assumed to be centered between the two ground planes. In this investigation, the thickness *b* is assumed to vary based on pressure being applied to the textile swatch when touched.

The characteristic impedance and propagation constant of a stripline are well established and are available in [23].

## 4. Simulation Results

To explore the potential capabilities of the three types of transmission lines, two types of simulations are performed. The characteristic impedances of the line in the touched and untouched state are compared. Ideally, there should be a large discrepancy between the touched and untouched state, resulting in a high-magnitude reflection coefficient at the touch interface. Given the characteristic impedances of the touched and untouched states, the reflection of the transmission line can be simulated for each location of a touch. We assume an operating frequency of 900 MHz, as a quarter-wave stub would be a convenient length to implement at this frequency.

### 4.1. Characteristic Impedances

The first set of simulations was performed to determine the sensitivity of the characteristic impedances of the transmission lines presented in Section 3.1 to changes in geometry (coax, stripline) or superstrate dielectric (microstrip). Such changes result from the wearer touching or manipulating the e-textile swatch.

#### 4.1.1. Coaxial Line

**Figure 9:**
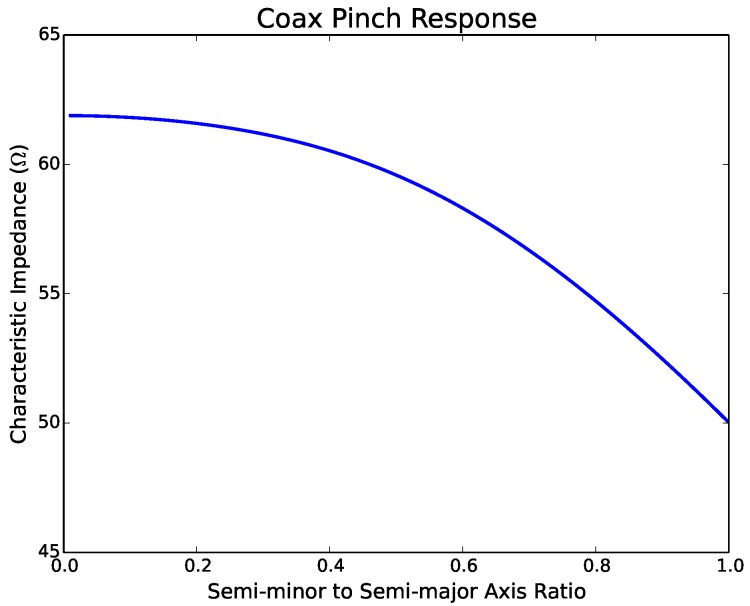
Effect of pinch on the characteristic impedance of the coaxial line.

To approximate the effect of pinching the coax, the coax was deformed to an ellipse whose semi-major and semi-minor axes are determined such that the area of the cross-section of the coax remains constant. Figure 9 shows the effect of pinching on the characteristic impedance of the coax. The characteristic impedance was calculated as a function of the ratio between the semi-minor and semi-major axes, where the characteristic impedance of the circular case is 50Ω. As the eccentricity of the cross-section increases, the impedance approaches ∼62Ω, which would result in a reflection coefficient of ∼0.107 at the location of the pinch.

#### 4.1.2. Microstrip

Simulating the effect of touching a microstrip involves determining the characteristic impedance of a microstrip with (i.e., touched case) and without (i.e., touched case) a dielectric superstrate. As this type of line involves direct contact with a human fingertip, the simulations are performed using realistic values for an e-textile microstrip. The dielectric layer of the e-textile microstrip consists of a 1.71-mm layer of denim and a conductive strip 6.35 mm wide and 0.08 mm thick. The relative permittivity of denim has been measured as $\epsilon_{r}=1.67$ [32]. Untouched, the microstrip has a characteristic impedance of 48.6Ω.

**Figure 10:**
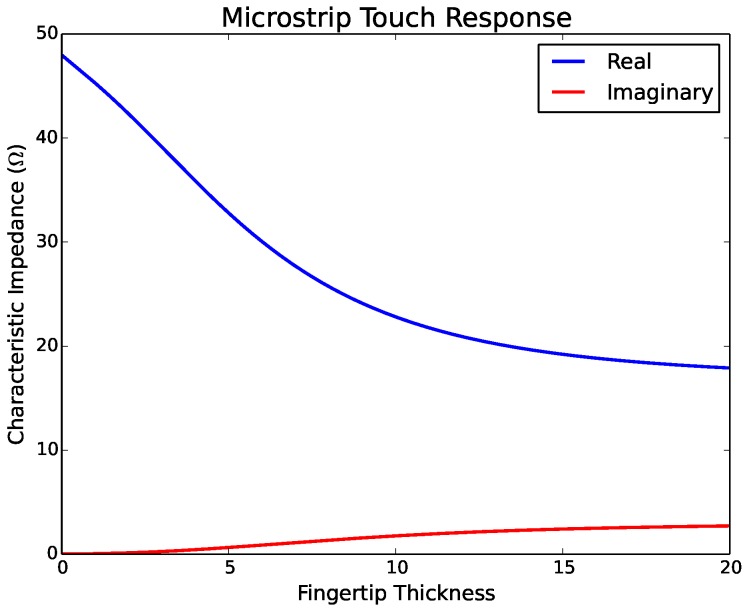
Effect of touch on the characteristic impedance of a microstrip line.

The dielectric properties of human skin have been measured in [33] over a wide range of frequencies. At 900 MHz, the relative permittivity of the palm is given as $\epsilon_{r}=44.5-j18.8$. From [34], the range of width of an adult human fingertip is given as 1.6 cm–2.0 cm. Figure 10 shows the characteristic impedance of the microstrip as a function of the thickness of the skin/fingertip touching the line. At fingertip thickness, the characteristic impedance is relatively constant: 19.2+j2.42Ω at 1.5 cm thick and 17.9+j2.72Ω at 2.0 cm thick. This corresponds to a reflection coefficient of $\Gamma =0.435∠173.3^{{}^{\circ}}$ and $\Gamma =0.463∠172.6^{{}^{\circ}}$, respectively, at the location of the touch.

#### 4.1.3. Stripline

**Figure 11:**
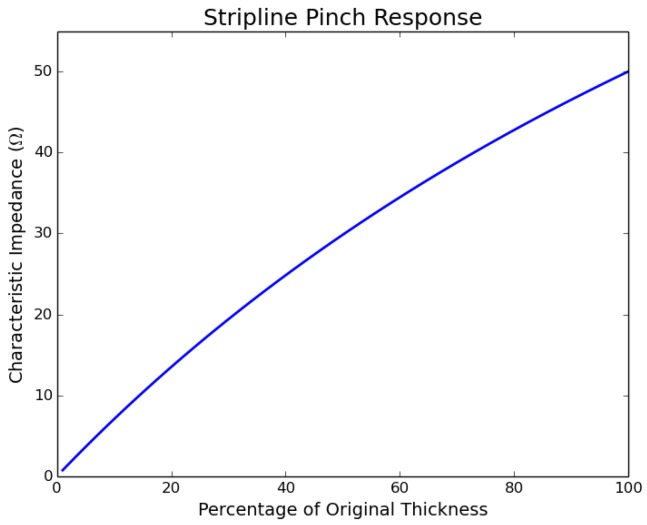
Effect of pinch on the characteristic impedance of a stripline.

Similar to the simulation performed on a coaxial line, simulations were performed to determine the effect of pinching or pressing on a stripline. The effect of pinching or pressing was simulated by reducing the original thickness of the stripline by a percentage of the original thickness. Figure 11 shows the characteristic impedance as a function of the percentage of the thickness of the original stripline, whose original characteristic impedance is 50Ω. The characteristic impedance at 75%, 50% and 25% of the original thickness is 40.83Ω, 29.84Ω and 16.51Ω, respectively, which corresponds to a reflection coefficient of ∼−0.100, −0.253 and −0.504, respectively, at the location of the pinch or press.

### 4.2. Reflection Coefficient

For each of the transmission line types, a simulation was performed where a quarter-wavelength short-circuit stub was pinched, touched or pressed at various locations. The simulations were performed at 900 MHz, corresponding to the frequency of operation of the designed reflectometer. A quarter-wavelength stub was selected, as it is assumed that each touch location would produce a unique reflection coefficient.

#### 4.2.1. Coaxial Line

A quarter-wave short-circuited stub made from a coax was simulated to show the effect of interacting with an e-textile swatch. A coax consisting of a center conductor made of a conductive thread with radius 0.21 mm, an outer conductor with radius 0.58 mm and a cotton dielectric, whose relative permittivity is 1.50 [32], has a characteristic impedance of 49.7 Ω and a propagation constant γ=j23.1. At 900 MHz, the quarter-wavelength stub is 6.8 cm in length.

The coax is simulated with semi-minor to semi-major axis ratios of 0.75, 0.5 and 0.25, corresponding to increases in pressure applied to the line. The corresponding characteristic impedances for these three ratios are 57.56 Ω, 61.43 Ω and 63.25 Ω, respectively.

**Figure 12:**
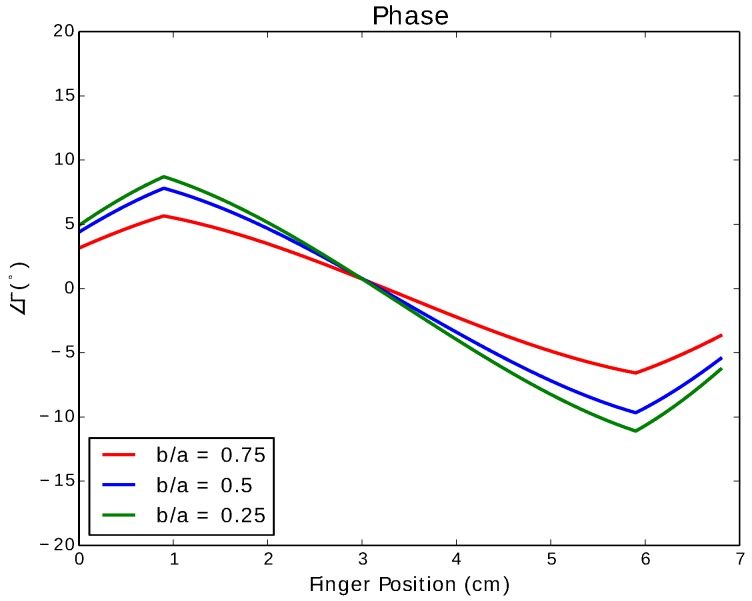
Phase of the reflection coefficient of a simulated quarterwave short-circuit stub using a coax.

Figure 12 shows the phase of the reflection coefficient of the coax when pinched as a function of the position of the pinch. As there are no losses in the line, the magnitude of the reflection coefficient remains at one. The fingertip is assumed to be 1.8 cm in width. The graph demonstrates a monotonic decrease in phase over a range of ∼$20^{{}^{\circ}}$ for the highest amount of deformation. Near the input port and shorted end, the fingertip does not fully cover the line, causing a reduction in the touch effect at either ends.

#### 4.2.2. Microstrip

A quarter-wave short-circuit microstrip stub was simulated using the cross-sectional geometry described in Section 4.1.2. At 900 MHz, a quarter-wave stub has a length of 6.8 cm. The characteristic impedance, effective permittivity and propagation constant of the microstrip line are summarized in Table 1 for the case where the line is untouched and touched by fingertips of widths from 1.6 cm–2.0 cm.

**Figure 13:**
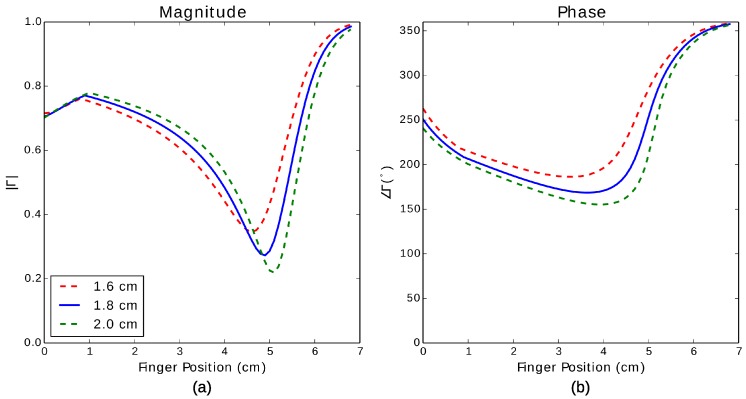
(**a**) Magnitude and (**b**) phase of the reflection coefficient of a simulated quarterwave short-circuit stub using a microstrip.

Figure 13 shows the magnitude and phase of input impedance of the line as a function of finger position on the line. The results demonstrate a consistent change in the reflection coefficient as the finger moves toward the shorted end of the stub; the interface between the touched and untouched portions has a high reflection coefficient, resulting in behavior similar to a shorted line increasing in length. At the shorted end, the high loss factor of the touched region acts as a load whose resistance decreases towards zero as the finger moves closer to the shorted end. These vary some from [8], where the touched region was more simply, though less accurately, modeled by assuming an infinite dielectric superstrate.

#### 4.2.3. Stripline

A third simulation involved the use of a stripline. Using a conductive strip with a width of 4.1 mm embedded in a cotton dielectric 5.0 mm thick, an untouched stripline has a characteristic impedance of 49.83 Ω. When pinching the line, compressing the stripline to 25%, 50% and 75% of the original thickness results in a characteristic impedance of 16.89 Ω, 30.19 Ω and 40.95 Ω, respectively.

**Figure 14:**
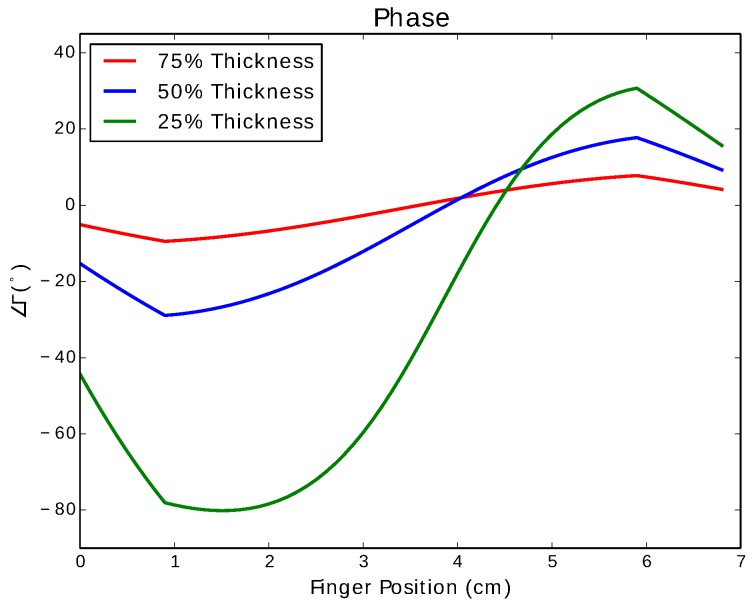
Phase of the reflection coefficient of a simulated quarterwave short-circuit stub using a stripline.

Figure 14 shows the phase of the reflection coefficient of the stripline when pinched as a function of the position of the pinch. The response is similar to a coax, though with a larger range in phase (∼100° for the largest amount of compression).

#### 4.2.4. Discussion

Based on the simulations, stripline and microstrip swatches are ideal for use as a gesture input device, as the coaxial line produces only a moderate amount of difference in the phase of the reflection coefficient based on where the line is pinched. While the phase of the reflection coefficient using a stripline has an appreciable range, a microstrip has two main advantages over this type of line. First, microstrips produce the largest variation in the phase of the reflection coefficient with respect to the finger position, as well as variation in magnitude. Second, the microstrip swatches are simpler to construct and integrate into existing garments. However, stripline swatches would be useful where a pressure-sensitive swatch is desired over a contact-sensitive swatch.

For touch input and gesture recognition purposes, a microstrip has the additional advantage of not relying on compression or deformation to detect contact. Thus, a stiff fabric whose thickness remains constant during deformation can be used to minimize the possibility of a false signal being generated. Additionally, the location of the sensor on the body can be selected to further minimize such noise, such as the forearm or thigh. Alternatively, the sensitivity to deformation of a stripline can be leveraged for alternative forms of input, such as detecting joint angles at the elbow or knee.

Selecting an appropriate fabric for insulation layers is an important engineering choice when constructing an e-textile swatch. One main concern is the stiffness and density of the fabric used. Coaxial and stripline swatches rely on a significant amount of compression; coupled with human touch forces in the range of 1–10 N [35], the insulating layers would require a low-density, highly deformable material, such as felt. For microstrip swatches, a denser material that does not deform when touched, such as denim, is ideal, as deformation would result in an unmodeled change in the characteristic impedance.

## 5. Hardware Implementation

### 5.1. Reflectometer Circuit

Evaluating the state of an e-textile swatch involves measuring the input impedance of the swatch using a reflectometer. A reflectometer measures the complex (magnitude and phase) reflection coefficient of a device under test, Γ, at the reflectometer’s measurement port, based on the reflectometer’s characteristic impedance, $Z_{0}$. Based on this measurement, the input impedance of the device under test can be calculated from Equation (3):

(5) $$Z_{in}=Z_{0}\frac{1+\Gamma }{1-\Gamma }$$

Accurate measurement of the magnitude and phase of the reflection coefficient typically requires an expensive vector network analyzer (VNA; e.g., Agilent HP 8510C, Agilent Technologies, Santa Clara, CA, USA). Several non-commercial devices have been developed [36,37,38,39], which perform the same operations. These devices are unsuitable for wearable devices for a variety of reasons: They use large, heavy waveguide components [39], require a scalar network analyzer [37], or use expensive phase shifter ICs [38]. Additionally, VNAs require a calibration set consisting of known loads in order to achieve accurate measurements.

An alternative approach to probing an e-textile swatch is to measure the relative change to the magnitude and/or phase of the reflection coefficient between untouched and touched states. While such a circuit would not be able to infer the location of a touch, a gesture may be determined by observing the change in signal over time.

**Figure 15:**
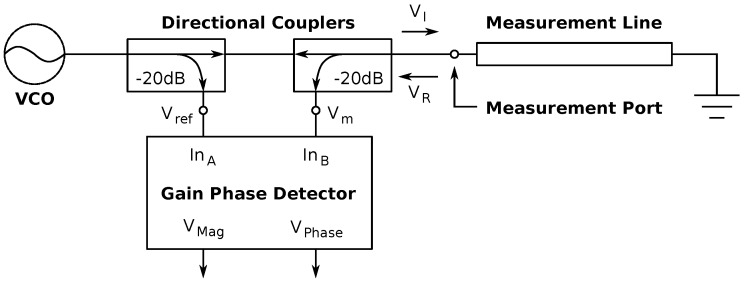
Schematic of the reflectometer circuit connected to SwitchBack.

A simple, inexpensive reflectometer circuit was built for this investigation. The circuit, shown in Figure 15, consists of a Maxim MAX2623 Voltage Controlled Oscillator (VCO) [40], two Skyworks DC09-73LF 20 dB directional couplers [41], an Analog Devices AD8302 gain phase detector [42] and a small number of passive components. The circuit transmission lines were designed to have a characteristic impedance of 50 Ω, matching the impedance of the components, and a female ultraminiature coax connector (UMCC) socket was used as a measurement port.

A 900-MHz signal is generated by the VCO at −3 dBm (500 μW). The first directional coupler splits a −23 dBm reference signal, $V_{ref}$, from the main signal, to be used by the gain phase detector. The signal is then passed through the second directional coupler and provides an input signal at the measurement port, $V_{L}$. A reflected signal, $V_{R}$, which is dependent on the input impedance of the swatch, is reflected back into the circuit. At the second directional coupler, the reflected signal provides a measurement signal, $V_{m}$, for the gain phase detector. The gain phase detector generates two DC voltages, $V_{Mag}$ and $V_{Phase}$, based on the reference and measurement signals, $V_{ref}$ and $V_{m}$. $V_{Mag}$ is calculated from the gain of the measured signal to the reference signal (|$V_{m}$|/|$V_{r}$|), while $V_{Phase}$ is calculated from the phase difference between the two signals ($∠V_{m}-∠V_{r}$). This combination of the two allows for measuring the coherent reflection coefficient.

In practice, the reflectometer and microcontroller circuits will be powered by a small battery, which will not share a common ground with the wearer’s body. The potential effects of a ground loop, however, are mitigated in the circuit in two ways. First, both the VCO and gain phase detector have capacitors at the signal and measurement ports, respectively, ensuring that any DC signals generated on the measurement line are decoupled from from the measured RF signal. Additionally, as a short-circuit stub is used as the measurement line, direct contact with the line results in a common ground being shared by the wearer’s body and circuit. Noise in the voltage measured at the ADC of the microcontroller can be accounted for by using a differential input if available. Noise may also be removed algorithmically by performing a measurement with the VCO placed in shutdown mode.

### 5.2. e-Textile Transmission Lines

A fabric microstrip swatch was constructed for experimental purposes, using a minimal number of materials and processes. The fabric swatch was designed by alternating layers of conductive and non-conductive fabric. The base layer, which serves as the ground plane for the microstrip line, was constructed from Rip-Stop conductive metalized nylon fabric [43] and measures 3.8 cm × 12.7 cm. A 2-mm layer of iron-on adhesive denim, sized to the same dimensions of the ground plane, constitutes the dielectric layer and was ironed directly to the conductive ground plane. The conductive strip consists of a 10 cm × 0.635 cm strip of Rip-Stop conductive fabric that is centered and adhered to the denim layer using fabric glue. On one end of the interface, 117/17 2-ply conductive thread [44] is used to sew through the swatch to connect the ground plane to the top layer, forming a short-circuit. On the opposite end of the interface, an ultraminiature coax line was attached. The outer conductor of the coax was soldered to the fabric ground plane, and the dielectric and center conductor were fed through a hole in the denim, where the inner conductor is soldered to the conductive strip. Soldering was performed using a low-temperature solder paste and a heat gun, to avoid warping the conductive nylon. This interface is designed to attach to a number of on-body clothing articles or other textile-based items.

## 6. Gesture Identification

Determining the absolute position of a finger on a microstrip swatch requires a reflectometer capable of being calibrated. For wearable applications, performing such a calibration would be difficult, and maintaining the calibration would be infeasible due to motion and variation in temperature. A more feasible approach is to identify specific gestures based on the temporal signal generated when a gesture is performed. This approach allows a simpler calibration approach where the reflection coefficient of the untouched line is coherently subtracted from future measurements.

### Convolutional Neural Networks

Convolutional neural networks (CNNs) are a type of feed-forward neural network that has been successfully used for classifying images and time series [45]. CNNs provide several advantages for classifying time series data, such as the gesture signals in this paper. CNNs automatically learn features from training data, rather than requiring hand-designed features. CNNs are also robust to scaling and shifting of features in the signal, making them robust to variations in speed of the gesture and the relative position of the finger on the microstrip.

**Figure 16:**
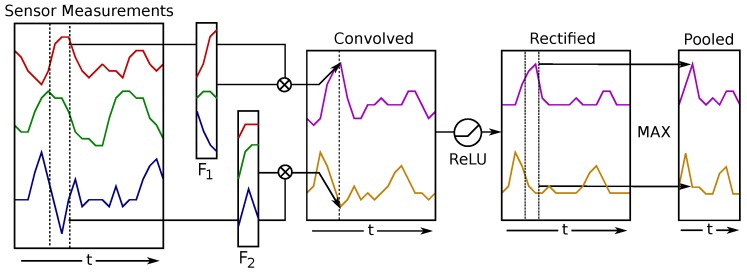
Example convolutional and pooling layer in the convolutional neural network. From [26].

CNNs consist of multiple sets of convolutional and pooling layers, as shown in Figure 16. The convolutional layer consists of multiple feature maps, $F_{n}$, which are convolved with the input signal. The *j*-th output signal of the *i*-th convolutional layer, $z_{j}^{\left(i+1\right)}\left(t\right)$, using the output signal from the *i*-th layer, $z^{\left(i\right)}\left(t\right)$, as input, is given by:

(6) $$z_{j}^{\left(i+1\right)}\left(t\right)=\sigma \left(b_{j}+\sum_{f=1}^{N_{F}}\sum_{\tau =0}^{l_{F}}F_{jf}^{\left(i+1\right)}\left(\tau \right)z_{f}^{\left(i\right)}\left(t-\tau \right)\right)$$

where $b_{j}$ is the *j*-th bias term, $F_{jf}$ is the *f*-th term of the *j*-th feature map, $l_{F}$ is the length of the feature maps and $N_{f}$ is the number of feature maps. A nonlinear function, *σ*, is applied to the output of the convolutional layer; a common function to use is the logistic function:

(7) $$\sigma \left(x\right)=\frac{1}{1+e^{-x}}$$

The pooling layer reduces the length of the output signal of the convolutional layer by summarizing a small pooling window. Max-pooling layers, which output the maximum value in the pooling window, are commonly used. The output of such a layer is given by:

(8) $$z_{j}^{i+1}\left(t\right)=max_{\tau =th}^{th+s}\left[z_{j}^{\left(i\right)}\left(\tau \right)\right]$$

where *s* is the pooling size (number of samples in the pooling window) and *h* is the stride (number of samples between the beginning of each pooling window).

Multiple convolutional/pooling layers can be implemented by using the output of one pooling layer as the input to a convolutional layer. This results in multiple layers, where higher layers summarize the signals into abstract representations. To perform classification, the final pooling layer is flattened and used as input to a softmax classification layer. The softmax layer produces a probability distribution given by:

(9) $$z_{j}^{\left(i+1\right)}=\frac{e^{z_{j}^{\left(i\right)}}}{\sum_{N}e^{z_{j}^{\left(i\right)}}}$$

where *N* is the number of labels in the distribution and $z_{j}^{\left(i+1\right)}$ is the probability that the signal is assigned label *j* by the network.

## 7. Experimental Results

Gesture detection and recognition experiments were performed to validate the performance of a microstrip e-textile prototype. The geometry and materials used for the prototype were those used in Section 4.1.2 and Section 4.2.2: the dielectric consisted of a 1.71 mm-thick layer of denim, and the conductive strip was 6.35 mm wide and 6.8 cm in length. For this, three different gestures were considered: up swiping, where the user slides his or her finger from the input port of the microstrip towards the shorted end, down swiping, where the finger is moved in the opposite direction, and tapping, where the finger briefly makes contact with one spot on the line. These low-level features may be considered a set of base gestures, which can be performed using a single finger, from which more complex gestures can be composed (e.g., double or triple tapping).

### 7.1. Sample Gestures

The three gestures were performed on the microstrip prototype to empirically demonstrate the ability to distinguish between the gestures. Additionally, the gestures were performed on three variations of the prototype; bare, covered with a cloth and soaked in silicone rubber (EcoFlex). The covered case allows for a discrete interface on a garment, while the silicone-soaked version provides weatherproofing.

**Figure 17:**
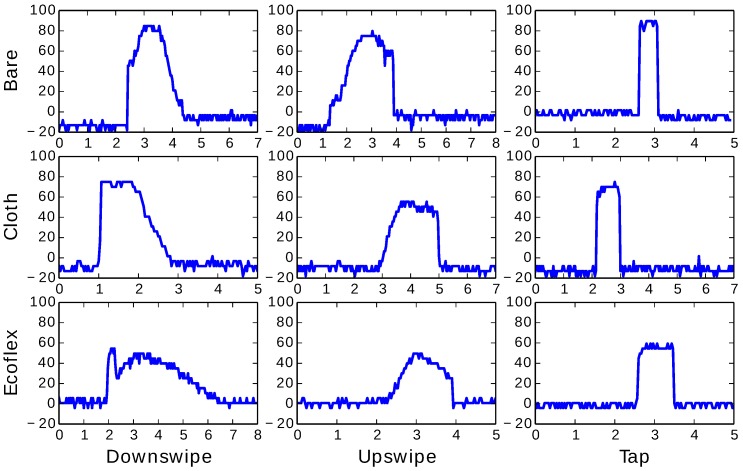
Characteristic gesture signals for down swiping, up swiping and tapping, for three configurations of the prototype. Horizontal axes are time (in seconds). Vertical axes are the measured voltage (in millivolts).

The voltage corresponding to the phase of the reflection coefficient was sampled at 30 Hz, with each sample being performed over the course of a few seconds. To calibrate, the reflection coefficient of the untouched line was coherently removed from the measured signal. Figure 17 shows the phase of the measured signal for each configuration and gesture. The signal (*y*-axis) is measured in millivolts, after coherent removal of the untouched reflection coefficient. Up swipes and down swipes produce an increasing or decreasing signal, followed or proceeded by a jump in the signal. Similarly, tapping results in a narrow pulse.

The characteristic signals vary little between the three variations. When embedded in EcoFlex, the dynamic range of the signal is reduced, likely due to the EcoFlex changing the permittivity of the denim. However, the profile of the signals remains unchanged, ensuring that the three gestures can still be distinguished.

### 7.2. Gesture Recognition

Gesture classification was performed using a convolutional neural network. A preliminary data stream was collected from a single subject. From the data stream, 60 tap gestures, 32 up swipe gestures and 26 down swipe gestures were collected and hand-labeled. The onset and conclusion of each gesture were indicated when the measurement signal was outside of the baseline signal by a threshold, which was determined empirically. The dataset was augmented by adding uniform noise with a range of 20% of the measurement range to the measurements, resulting in 120 samples of each gesture. This ensured an even distribution of the gestures in the dataset and ensured that the CNN would not be overfit to the training data. The reflection coefficient phase was only used, as this was determined to be sufficient for classification purposes and reduced the size of the CNN.

Each gesture was captured in a window of 50 samples (∼1.67 s of measurement), which was sufficiently large enough to contain each gesture. A CNN was trained to classify the gestures and consisted of two convolutional/max-pooling layers, a fully-connected layer and a softmax layer. The first convolutional layer had a window size of 10 samples and five feature maps. The second convolutional layer had a window size of five samples and three feature maps. Both pooling layers had a pooling size and stride of four samples. The output of the second pooling layer consisted of six values, and the fully-connected layer contained 12 units. The logistic function, given in Equation (7), was used as an activation function for both convolutional layers and the fully-connected layer. The number of parameters required for this model, which directly correlates to the memory and computing requirements, is 256. Using 32-bit values, this requires only 1 kB of flash memory on a microcontroller to implement, allowing for a very inexpensive microcontroller (e.g., AVR ATtiny44A, Atmel, San Jose, CA, USA) to be used for data collection, processing and communication.

The CNN was implemented using TensorFlow [46] and trained using the Adam optimizer [47], with a learning rate of 0.005 and all other parameters set as those suggested in [47]. The performance of the model was estimated using 10-fold cross-validation, which allows for better estimates of performance when using small datasets. The final model accuracy is estimated to be 96.11%. Table 2 shows the confusion matrix of the trained model, showing that misclassifications occur primarily with tap gestures.

## 8. Conclusions

In this paper, we explore the use of RF-based, e-textile input devices for gesture recognition in garments and other fabric-based objects. These swatches provide eyes-free, one-handed interfaces capable of handling multiple types of input. Our approach provides several benefits over similar tactile input devices based on resistive, capacitive or circuit completion approaches. Swatches are easily manufactured, can measure a continuum of finger positions using a single measurement line and do not require direct skin contact to perform correctly, allowing swatches to be covered or weatherproofed. Three potential swatches were compared based on transmission lines common in RF and microwave engineering: coaxial lines, microstrips and striplines. From the simulations, we note that microstrips are an ideal approach, as these provide a large dynamic range in both the magnitude and phase of the reflection coefficient with respect to finger position. Striplines are similarly sensitive with respect to the phase of the reflection coefficient and provide a sensing modality relying on pinching or pressing rather than contact or near-contact, whereas coaxial lines are relatively insensitive to pinching or pressing.

Experimental results with a prototype microstrip swatch demonstrate that characteristic signals for common low-level gestures (tapping and bi-directional swiping) can be distinguished from each other and are not affected by a thin fabric covering or weatherproofing. Additionally, we show that a small CNN can classify these gestures with an accuracy of 96.11%. Additionally, measurement, classification and communication to external devices can be achieved with a small, inexpensive microcontroller.

There are several possible avenues for future investigations. Using a reflectometer capable of measurements over a wide frequency band would allow for precise determination of finger position, as well as detecting multiple contact points. This would also enable the implementation of two-dimensional microstrip swatches, which could provide similar functionality as a smartphone touchscreen. Furthermore, more complex gestures could be identified from sequences of the low-level gestures presented here, using hidden Markov models or finite state machines, allowing the user a larger number of potential interactions. Finally, ongoing miniaturization of electronic components has allowed us to integrate high-frequency sensing with non-trivial signal processing, paving the way for a new generation of smart composites not limited to gesture recognition in textiles, but non-destructive evaluation or touch-sensitive robotic skins that require little to no external computation.

## Figures and Tables

**Table 1:** Transmission line parameters for a touched and untouched microstrip.

Fingertip Width	$Z_{0}\left(\Omega \right)$	$\epsilon_{\mathrm{eff}}$	*γ* (rad/m)
Untouched	48.56	1.50	0.0+j23.10
1.6 cm	18.84+j2.50	9.54−j2.57	7.78+j58.78
1.8 cm	18.28+j2.62	10.04−j2.62	8.60+j60.38
2.0 cm	17.90+j2.72	10.40−j3.23	9.34+j61.54

**Table 2:** Confusion matrix of classified gestures.

		Predicted		
		Tap	Up Swipe	Down Swipe
**Actual**	Tap	**113**	3	4
	Up swipe	1	**118**	1
	Down swipe	4	1	**115**
